# A Novel Echocardiographic-Based Classification for the Prediction of Peri-Device Leakage following Left Atrial Appendage Occluder Implantation

**DOI:** 10.3390/jcm11041059

**Published:** 2022-02-18

**Authors:** Ali Hamadanchi, Shun Ijuin, Franz Haertel, Tarek Bekfani, Julian Westphal, Marcus Franz, Sven Moebius-Winkler, P. Christian Schulze

**Affiliations:** 1 Division of Cardiology, Angiology and Intensive Medical Care, Department of Internal Medicine I, University Hospital Jena, Friedrich-Schiller-University, 07747 Jena, Germany; silver.gray.dragon@gmail.com (S.I.); franz.haertel@med.uni-jena.de (F.H.); julian.westphal@med.uni-jena.de (J.W.); marcus.franz@med.uni-jena.de (M.F.); sven.moebius-winkler@med.uni-jena.de (S.M.-W.); christian.schulze@med.uni-jena.de (P.C.S.); 2 Department of Cardiology, National Hospital Organization Kagoshima Medical Center, Kagoshima 892-0853, Japan; 3 Division of Cardiology, Angiology and Intensive Medical Care, Department of Internal Medicine I, University Hospital Magdeburg, Otto von Guericke-University, 39120 Magdeburg, Germany; tarek.bekfani@med.ovgu.de

**Keywords:** left atrial appendage, percutaneous closure, para device leakage, echocardiography

## Abstract

(1) Background: The assessment of residual peri-device leakages (PDL) after left atrial appendage occlusion (LAAO) remains crucial for post-procedural management. Our study aimed to verify a novel echocardiographic classification for the prediction of PDL. (2) Methods: Echocardiographic data of 72 patients who underwent percutaneous LAAO were evaluated. All echo images were analyzed by two independent investigators using standard analysis software (Image-Arena IA-4.6.4.44 by TomTec^®^, Munich, Germany). A total number of 127 studies was evaluated. Forty-four patients had baseline studies, at 45 days and at 6 months post-implantation. We propose a morphological classification of LAA devices based on the amount of echodensity inside the devices into three types: type A showing complete homogenous thrombosis, type B incompletely thrombosed device with inhomogeneous echo-free space <50% of device, and type C with partially thrombosed device in which the echo free space was >50% of device in various planes, which we called the “ice-cream cone” sign. Each type was matched to the degree of PDL and clinical outcome parameters. (3) Results: Patients with type C had the highest percentage of PDL at 45 days follow-up (type A: 24%, type B: 31%, type C 100% PDL, *p* < 0.001) and at 6 months follow-up (type A: 7%, type B: 33%, type C 100% PDL, *p* < 0.001). Notably, device size in patients with PDL was larger than that in patients without PDL at 6 months follow-up (25.6 ± 3.5 mm vs. 28.7 ± 3.4 mm, *p* = 0.004). Device size in patients with type C appearance was the largest of the three types (type A: 25.9 ± 3.6 mm, type B: 25.8 ± 3.4 mm, type C 29.8 ± 3.0 mm, type A vs. C; *p* = 0.019; type B vs. C, *p* = 0.007). (4) Conclusions: In conclusion, PDL are common post-LAAO, and their frequency is underestimated and under-recognized. PDL are much more common in patients with larger LAA ostial sizes and likely lower longitudinal compression. Type C appearance of the LAAO devices (“ice-cream cone sign”) has a high positive predictive value for PDL. Further studies are needed for better delineation of the clinical importance of this proposed classification.

## 1. Introduction

Left atrial appendage occlusion (LAAO) has been increasingly regarded as an important alternative to oral anticoagulation for stroke risk reduction among patients with atrial fibrillation (AF) who cannot tolerate the oral anticoagulation therapy [1,2]. 

Multimodality imaging has developed and is established for device sizing and optimizing procedural success, which relies on complete closure of the left atrial appendage (LAA) [3]. The diversity in anatomy and shape of the LAA makes the implantation challenging. Therefore, peri-device leaks (PDL) can occur in a number of patients [4,5,6,7,8]. Moreover, a precise definition, pathophysiologic mechanisms, and optimal management especially for those who cannot tolerate long-term anticoagulation have not been established [4,6]. 

PDL of any degree occurs in approximately 12.5–32% of patients 12 months post-implantation as detected by transesophageal echocardiography [6,7,8]. It is important to consider that the definition of severe or significant PDL most commonly refers to a defect size of more than 5 mm diameter between device edge and LAA tissue on TEE color Doppler interrogation using a Nyquist limit set around 25 cm/s. Using this definition, significant PDL occurs in 5–20% of patients after endovascular or epicardial closure [4,9,10]. The accurate assessment of residual leaks remains crucial in the decision-making with regard to the best post-procedural management [11].

TEE is considered the standard imaging modality for the imaging of LAA before, during, and after implantation [2,10]. However, its accuracy is mainly determined by the quality of imaging [7]. The crescent-shaped anatomy of PDL makes detection and measurements challenging. Consequently, it is not surprising that TEE occasionally fails to detect minor or even major leaks [10].

Cardiac CT angiography (CCTA) appears to be a feasible alternative to TEE for post-LAA device surveillance to evaluate for device thrombus, residual leak, embolization, position, and pericardial effusion [12], but it is costly and not widely available in comparison to TEE. The diagnostic accuracy of leak detection by CT is unclear and requires further studies. 

We observed that on follow-up TEE evaluations of LAAO, certain recognizable patterns of LAA devices were identifiable. We specifically analyzed our single center dataset for the incidence and clinical relevance of PDL and its correlation to a novel classification based on echocardiographic features and its diagnostic accuracy for predicting PDL. Further, we analyzed predisposing factors for PDL and the role of BNP as a biomarker in predicting PDL post-LAAO.

## 2. Methods

We retrospectively evaluated echocardiographic data of patients who underwent percutaneous LAAC in Jena University Hospital between September 2015 and September 2018. All patients gave written informed consent. The local IRB approved this study. 

### 2.1. LAAO Procedure

The implantation of LAAO devices underwent using a standard procedure [13]. The LAA closure device was implanted under conscious sedation and TEE control in the catheterization laboratory as previously explained in detail [14]. 

After implantation, patients remained on aspirin 100 mg and either NOACs or Vitamin K antagonists (internal normalized ratio 2.0–3.0) for 45 days post-implantation. At 45 days, TEE was performed to assess the device placement to assess whether the LAA was closed completely and thrombus on the device was ruled out. 

When flow through leakage was noted around the device >5 mm, consideration was given to keep the patient on anticoagulation until it had decreased to <5 mm. Patients ceasing anticoagulation were started on clopidogrel 75 mg and aspirin daily for 3–6 months post-implantation and continued taking aspirin daily. 

In some cases, aspirin was finished at 12 months at the discretion of the physician. In patients with impaired renal function, enoxaparin was prescribed for 4 weeks without using OAC and replaced by aspirin and clopidogrel for 3 months, and aspirin was continued at 12 months [14].

In all patients we included in these data (*n* = 72), the Watchman Occluder (Boston Scientific, Marlborough, MA) was implanted, and we excluded all devices other than Watchman to avoid statistical inconsistencies.

### 2.2. Transesophageal Echocardiography

Transthoracic echocardiography (TTE) and TEE examinations were performed routinely in all patients at baseline during the procedure and following 45 and 180 days, using an ultrasound system (IE33 or EPIQ, Philips Healthcare, Andover, MA, USA). The recordings were processed using a dedicated software (Image-Arena™ Version 4.6; TomTec Imaging Systems, Unterschleissheim, Germany). The detailed methodology of acquisition and analysis of the LAA before and after procedure was previously reported by our group [15].

**Figure 1:**
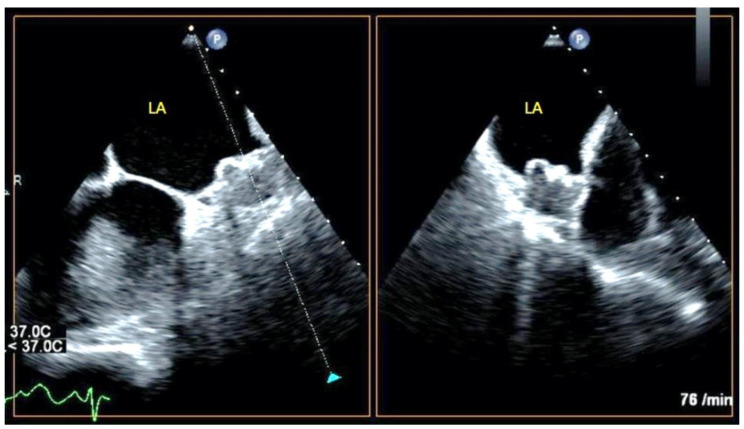
TEE demonstration of a type A device in two orthogonal planes showing no echo-free space.

**Figure 2:**
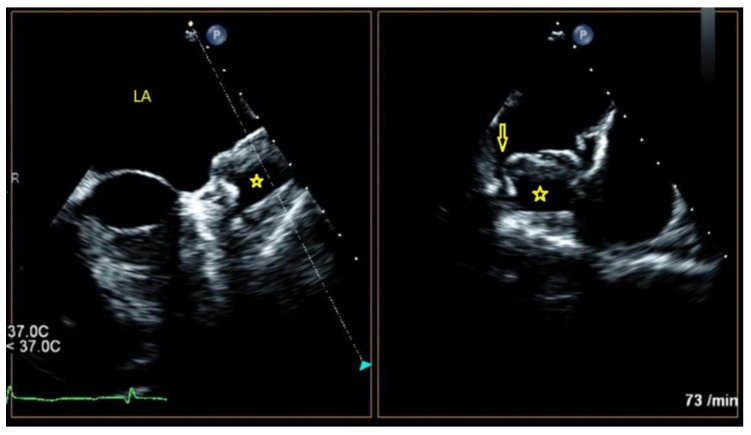
TEE image of another LAA device after 6 months of implantation showing almost 50% thrombosis within the device. * indicates the echo-free space below the thrombus. Arrow shows the PVL.

**Figure 3:**
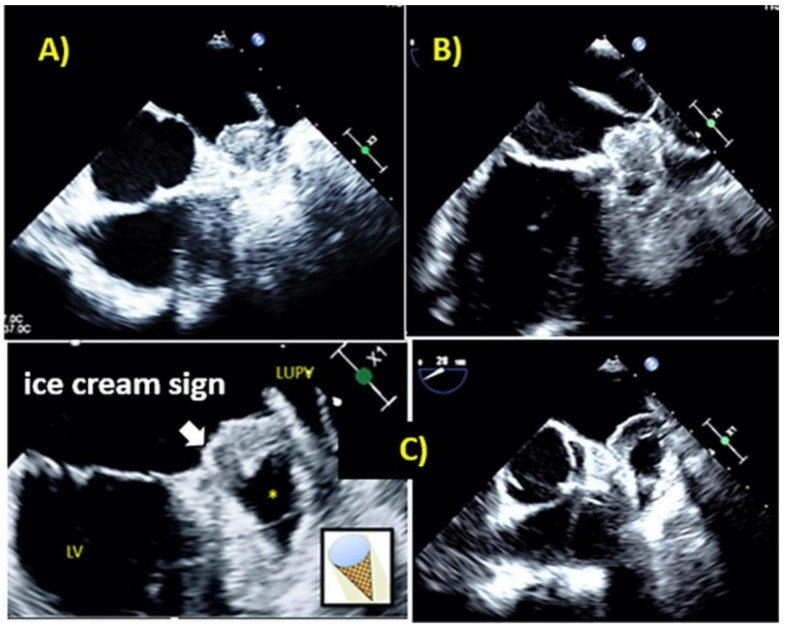
(**A**–**C**) All three types in an image. Left lower panel shows the ice cream cone sign. Type A: completely thrombosed device with complete homogenous echodensity inside the implanted device on standard views (0–135°) or using the X-plane mode with scanning through the device. Type B: incomplete thrombus formation inside the device with echo-free area (*) of less than 50% of device area in different views. Type C: partially thrombosed device, in which echo-free “black” areas comprised roughly more than 50% of total device area at least in two orthogonal views, the “ice-cream cone” sign due to its appearance on TEE images.

Using these data, we proposed a morphological classification of LAA occluder device features following implantation based on our previous observations and the degree of thrombus formation inside the device (Figure 1, Figure 2 and Figure 3). 

**Figure 4:**
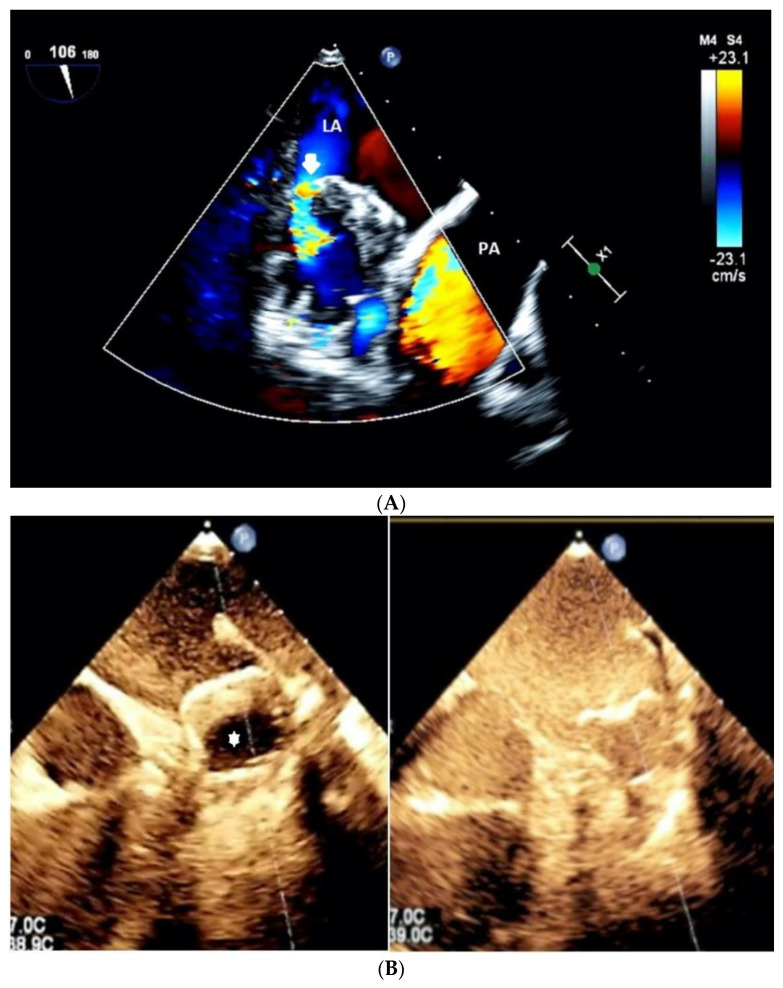
(**A**,**B**) A Color Doppler interrogation (**A**) and Ultrasound-enhanced imaging using SonoVue injection of the LAA device. Arrow signifies the inflowing blood flow inside the LAA through the PDL, explaining the pathophysiology of type C lesion. In (**B**), the echo-free area (*) of the device was filled completely after implication of contrast study.

**Figure 5:**
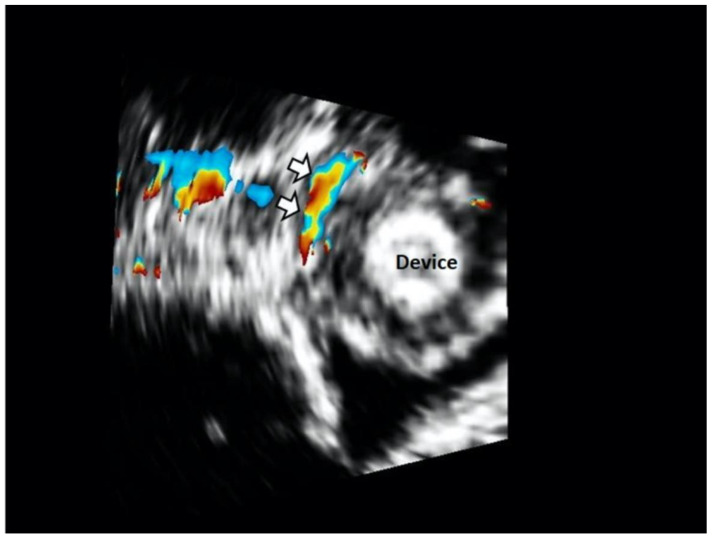
A 3D color Doppler en face view of the device of a patient with type C lesions demarcating the crescent-shaped nature of the leakage (white arrows).

We speculated that Type B represents an intermediate stage between complete and incomplete thrombus formation within the device, a transitional stage capable of moving to both directions based on device-host interactions and amount of PDL. Using color Doppler interrogation and Ultrasound enhancing agents, one can easily observe the blood flow inside the device and LAA (Figure 4 and Figure 5 and Videos S1–S3).

Echocardiographic evaluation of PDL was undertaken at least in two orthogonal views with color flow Doppler as described elsewhere by one of the investigators [3]. The data were analyzed separately by two investigators and were finally verified by one experienced echocardiography specialist (A.H.). Each patient was classified according to the degree of PDL and other echocardiographic, clinical, and para-clinical parameters. 

### 2.3. Biomarker

Blood samples were collected immediately before and 45 days and 6 months after LAAC. All measurements of brain natriuretic protein (BNP) were performed by using a chemiluminescent magnetic microparticle immunoassay (ARCHITECT i2000SR analyzer, Abbott, Chicago, IL, USA) at the Institute of Laboratory Diagnostics, University Hospital Jena. 

### 2.4. Statistical Analysis

Statistical data analysis was performed using SPSS Statistics (version 27.0, SPSS Inc., IBM, Armonk, NY, USA). Normal distribution was analyzed by the Kolmogorov–Smirnov test. Differences in the frequency of nominally scaled parameters were compared by means of Pearson‘s chi-squared test. Metric variables are expressed as mean ± standard deviation; tests on differences were made by Student’s *t*-test for independent and dependent variables. Binary logistic regression models were used to quantify the predictive value of the proposed model for the respective types. To specify the result of binary data in logistic regression, the block 1 method including an omnibus tests of model coefficients together with a Hosmer and Lemshow test were used. Correlation analyses were performed using Pearson’s test for pure metric values and Kendall‘s taub’s Spearman’s test for mixed correlations (nominal, ordinal, or metric values). Other models for prediction were generated by using crosstabs based on the 45 days and 6 month results of the type allocation; comparison took place with Pearson’s chi-squared test. Receiver operating characteristic (ROC) curves were generated, and their respective area under the curve (AUC) values are described. The basis for the test decision was a significance level of *p* < 0.05.

## 3. Results

### 3.1. Patient Characteristics

We included 72 patients in this analysis. A total number of 127 studies at various time points were evaluated. Forty-four patients had complete baseline studies as well as 45 days and 6 months follow-up post-implantation (Table 1). None of the patients died during the procedure or during follow-up. Two patients developed a hemorrhagic stroke. Device-related thrombosis was found in four cases, two of them in type C and two in type A. Two pericardial effusions also developed following the procedure. 

### 3.2. Echocardiographic Features Following LAAO

The mean 3D-area-derived LAA diameter was 19.8 ± 4.1 mm, and the mean device diameter was 27.36 mm.

At the 45 day follow up, 51% of cases were classified as Type A and 22% and 27% as Type B and Type C, respectively. PDL was noted in 37% of patients after 45 days.

**Figure 6:**
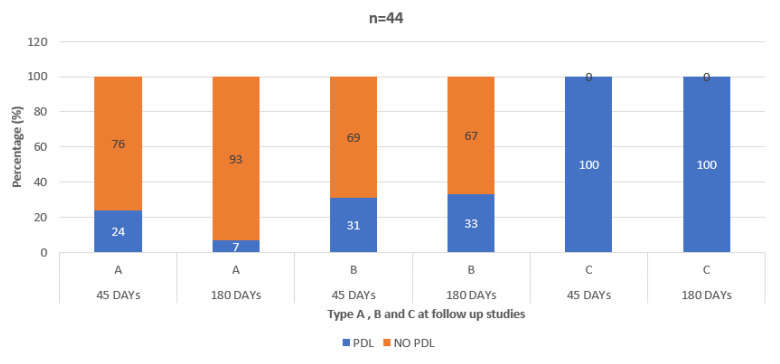
Peri-device leakage in each morphological category after 45 and 180 days.

Patients with type C had the highest percentage of PDL at 45 days follow-up (type A: 24%, type B: 31%, type C 100%, *p* < 0.001). Similarly, patients with type C had the highest percentage of PDL at 6 months follow-up (type A: 7%, type B: 33%, type C 100%, *p* < 0.001) (Figure 6).

The device size in patients with PDL was larger compared to patients without PDL at the 6 months follow-up (25.6 ± 3.5 mm vs. 28.7 ± 3.4 mm, *p* = 0.004). The device size in patients with type C was the largest of the three types (type A: 25.9 ± 3.6 mm, type B: 25.8 ± 3.4 mm, type C 29.8 ± 3.0 mm, type A vs. C, *p* = 0.019; type B vs. C, *p* = 0.007).

The orifice area in patients with type C was the largest of the three types at the 6 months follow-up (type A: 2.9 ± 1.1 cm^2^, type B: 3.0 ± 0.8 cm^2^, type C 4.2 ± 1.3 cm^2^, type A vs. C, *p* = 0.027; type B vs. C, *p* = 0.028).

We could not identify significant differences in compression strength at 6 months follow-up between the three types (type A: 13.6 ± 6.7%, type B: 12.3 ± 7.9%, type C: 12.5 ± 5.9%, type A vs. C, *p* = 0.887; type B vs. C, *p* = 0.998).

Regarding the PDL size, immediately after the procedure, PDL was noted in 19/127 patients or 15%. In each morphological category, the mean diameter for type A (*n* = 2) was 2.5 mm and for type B (*n* = 11) and type C (*n* = 24) were 2.78 mm and 3.8 mm, respectively (*p* = 0.142).

### 3.3. Predictors of Type C LAAO

LAA orifice area at baseline showed the strongest AUC for prediction of type C occlusion (AUC 0.78, *p* = 0.019) (Table 2 and Table 3). The implanted occlude device has shown good correlation for the prediction of type C and PDL (OR 1.23, *p* = 0.023). The sensitivity of “type C” appearance of LAA in predicting the “overall” leak at the 45 day TEE was 100% (positive likelihood ratio: 2.7; Table 4) with a specificity of 63%. On the other hand, type A disclosed a 100% negative predictive value for a relevant PDL. In other words, type A morphology speaks against a PDL based on our data.

### 3.4. Correlation of BNP Values with Morphological Features 

**Figure 7:**
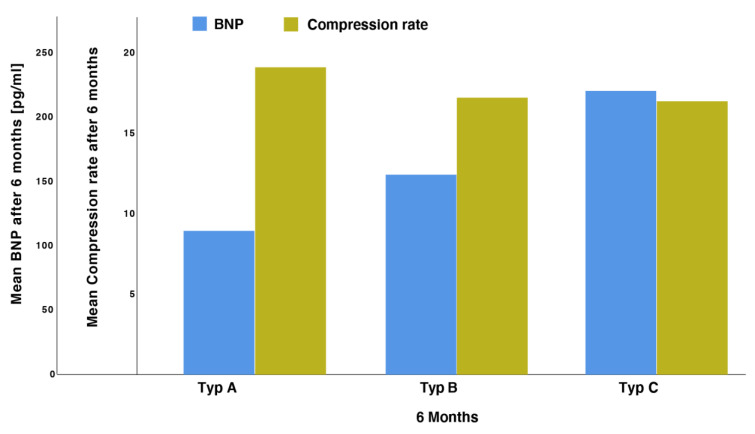
Levels of BNP in patients with various echocardiographic types.

Our data showed that the patients with the highest compression rate at 45 days and 6 months had the lowest values of BNP at baseline. Type C patients showed the highest baseline values of BNP in comparison to Type A patients (Figure 7).

## 4. Discussion

The current study describes the frequency and features of PDL following LAAO in patients with atrial fibrillation at intermediate risk for thromboembolic events. We here describe a new echocardiographic finding of distinct imaging appearance (“ice cream cone sign”) with high predictive value for the presence of a large PDL.

The main findings of the study are the unexpectedly high incidence of PDL at 6 months (41% of all patients). Our proposed classification based on echocardiographic appearance allows the discovery and quantification of PDL even in the absence of classical color Doppler imaging. Almost all patients with type C PDL at 6 months according to our classification showed a high degree of PDL. Type C PDL was positively and statistically significantly correlated to LAA ostial size and was linked to lower lateral device compression. Finally, BNP serum levels remained elevated following LAAO among the type C PDL patients, while levels in all other groups decreased over time. 

Percutaneous left atrial appendage occlusion (LAAO) is regarded as an alternative for stroke prevention in patients with non-valvular atrial fibrillation in whom anticoagulation is absolutely or relatively contraindicated [1]. Several large, randomized trials and associated meta-analysis have shown to date that LAAO is non-inferior and in some high-risk populations even superior to anticoagulation therapy [11,16,17,18,19]. Like other procedures, LAAO has limitations concerning patient selection and device selection due to anatomical features of the patient. One of the most important challenges is the incomplete closure of the LAA resulting in PDL and inefficient protection from thrombi formation and thus incomplete protection from thromboembolic events [1,9,20]. 

The assessment of residual PDL after LAAO remains crucial in the decision-making regarding post-procedural management and is part of routine follow-ups of patients post-LAAO. Incomplete LAAO may create a small “pocket” containing thrombus risking systemic embolization. Residual flow resulting from incomplete closure within the device may promote local stagnation of blood flow and thrombus formation and subsequent systemic embolization [7,8]. It is believed that adequate closure of LAA requires appropriate neo-endothelialisation of the device, and this process will not happen in the absence of defects or leaks. Thus, accurate recognition of PDL is an increasingly recognized problem following LAAO. Small residual shunts with a jet diameter < 5 mm are usually deemed irrelevant and may close spontaneously over time. 

In the past, criteria for successful closure of the LAA have ranged from <1 mm up to <5 mm residual flow into the LAA on color Doppler flow [10]. Of note, two large randomized trials of LAAO using the Watchman device have both used the arbitrarily defined <5 mm residual flow as success criteria for LAAO [7,9,10,21,22], while others using the Amplatzer™ Cardiac Plug (ACP) [23] and Amulet device have reported leaks > 5 mm as significant and 1–3 mm as moderate [6]. With the Watchmen device, any PDL on TEE was observed in about 32% of patients at one year (PROTECT) [8]. 

In a sub-study of the Watchman PROTECT AF (Percutaneous Closure of the Left Atrial Appendage Versus Warfarin Therapy for Prevention of Stroke in Patients With Atrial Fibrillation) trial, investigators detected “major” PDL defined as >3 mm in 32.4% of patients but did not observe any association with PDL and an increased risk of thromboembolism [8,9].

A few small reports of stroke related to smaller leakages have also been published [7,8]. It has not been clarified whether PDL is related to device-related thrombosis (DRT) or not, possibly due to the small size of studies, but DRT is mostly found on the central part of the device, a place far from PDL [24]. In a study by Pracon et al., neither device type nor postprocedural peridevice leaks were related to DRT [25].

It should be noted that incomplete occlusion does not necessarily imply to PDL. A persistent large accessory lobe adjacent to LAA that remains unclosed by the occluding device can also cause thromboembolism [24]. This problem is more common for devices that utilize the “plug principle,” such as Watchman occluders. Devices that use the “pacifier principle,” with proximal disc, can cover this accessory lobe and, therefore, have most likely a reduced incidence of thromboembolism [26]. 

Furthermore, the PDL is not limited to the interventional devices. Of note, surgical LAA closure resulted in 20–40% of residual defects at follow-up imaging, and this was associated with higher risk of stroke and systemic embolization compared to those with complete closure of LAA [27,28]. Whether persistent large (>5 mm) shunts deserve long-term OAC with VKA or NOAC or a second occlusion attempt remains at the operator’s discretion [1]. Therefore, the exact diagnosis, natural history, and optimal management of PDL remains a common clinical dilemma [9,21,29,30].

Initial reports of small PDL (jet width ≤ 5 mm) 1 year after Watchman implantation were as high as 32% in PROTECT-AF [8], but more recent studies (PREVAIL and EWOLUTION) reported <10% incidence [16,18,31]. It has been suggested that PDL depends on the interaction between LAAO devices, anatomical characteristics of the LAA, and possibly distinct tissue characteristics [9]. 

Cardiac-computed tomography angiography (CCTA) due to the use of contrast agents without definition of flow characteristics in the LAA is more sensitive for detection of PDL, while its specificity for the degree of PDL is lower [12,29,30]. It is, therefore, not surprising that the frequency of PDL post-LAAO detected by CT has been described to be as high as 50–60% [9,12,32]. The predictors of PDL after LAAO are not clearly delineated, although one report has shown that a device compression rate less than 10% is associated with PDL [29]. 

Our study patients also had a surprisingly high incidence of PDL 6 months after LAAO. We suspect that this possibly reflects the high sensitivity of color Doppler interrogation methods. Further, our data were related to one single type of LAAC device. 

The complex anatomy of the LAA and the pathophysiology of PDL requires not only a sophisticated assessment of the LAA but also a practical echocardiographic classification that also considers the three-dimensional and elliptical geometry of leaking orifices of PDL. The exact definition of leakage severity in terms of the echocardiographic diagnosis and the long-term clinical impact is still poorly defined and understood. Whether the PDL or peri-leaks are associated with increased risk of stroke or embolic phenomena is undefined. 

**Figure 8:**
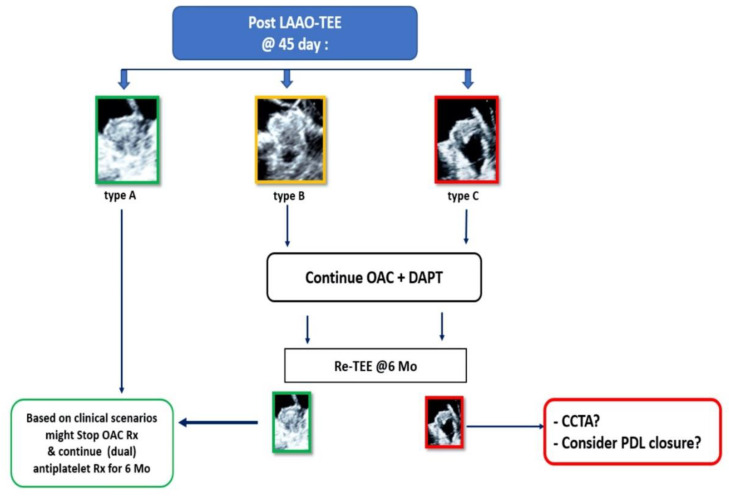
Our proposed approach for the management of LAAO based on morphological classification. LAAO-TEE = Left atrial appendage occlusion transesophageal echocardiogram; OAC = Oral anticoagulation; DAPT = Dual antiplatelet therapy; PDL = Peri-device leakage; CCTA = Cardiac-computed tomography angiography.

We here propose a new morphological classification of the LAA device morphologies following implantation based on echocardiographic features related to the amount of internal thrombus formation. Type B represents an intermediate stage, which can result in complete closure (type A) or remains open without complete thrombus development allowing blood flow within the incompletely thrombosed the device. This simplified classification might potentially lead to a practical approach (Figure 8) for the management of LAAO in everyday practice. Further verification though is required. 

Our data are in concordance with other studies who also used echocardiographic features to classify the appearance of the LAA after LAAO into white (W) and non-white (NW) based on imaging parameters [5]. The NW group, which corresponds to our type B and C, was a predictor of a significant leak also in our analysis (OR: 47.96, CI: 2.91–790.2, *p* = 0.007) [5].

Taking problems of echocardiographic recognition of PDL into consideration, our study is an attempt to improve the classification of inadequate device closure of the LAA. Instead of measuring color Doppler-derived gaps in follow-up echocardiographic assessments that are prone to measurement failures due to elliptic nature of defects, we here propose a new classification system. Further, we demonstrated that the ostial size correlated well to the development of PDL. 

Ultimately, the definition of “severe” or clinically significant PDL remains to be defined as no definitive correlation to the adverse outcomes has been demonstrated yet. The associated pathophysiologic mechanisms are also to be accurately outlined; however, morphological diversity in LAA ostium and body, and most likely individual tissue characteristics and fibrotic remodeling, will play a major role. 

We also demonstrated a correlation between absolute BNP values and their dynamics over time and type C classification. We speculate that residual defects allow blood flow inside the appendage and thus prevent “shrinkage” and inactivation of secretory pathways of the LAA tissue resulting in prolonged hypersecretion of BNP. The inverse relation of BNP values with LAAO was evaluated previously [33]. Our current results suggest that percutaneous closure of the LAA results in an intermittent distribution of vasoactive hormones ANP and BNP followed by a significant attenuation of ANP and BNP secretion in the early post-procedural period. The clinical impact of these findings and the potential role of cardiac biomarkers for the prediction of procedural success and post-LAAO complications needs to be evaluated in further studies [33].

### Limitations

We acknowledge several limitations in our study. First, we retrospectively analyzed all data of 72 consecutive patients with all limitations of retrospective observational studies including selection bias and inadequacy and incompleteness of full information in some patients. Second, PDL was diagnosed only by TEE without systematic comparison with CCTA. Since our retrospective study did not include CCTA imaging, the exact morphological classification of LAA morphologies was not amenable [34]. Third, the dataset was established on data from a single center experience with a relatively small number of patients with only one device type. This might indicate that this classification might be only applicable to patients following endocardial Watchman LAA occlude device placement since we did not have a sufficient number of other devices included in our analysis. Fourth, the clinical outcome of each echocardiographic type was not included.

## 5. Conclusions

In conclusion, we here proposed a novel classification system based on standard echocardiographic assessments of LAA appearance following LAAO. Based on our data, type A or a completely thrombosed device virtually excludes a significant PDL, while type C is strongly suggestive. We defined high-risk features for severe PDL and proposed a novel echocardiographic imaging finding associated with severe PDL (“ice cream cone sign”). Further, we showed the association of PDL with anatomical features of the LAA and the predictive value of the dynamics of BNP levels post-LAAO. 

## Figures and Tables

**Table 1:** Baseline demographic and clinical characteristics of the total study population.

	Study Population (N = 72)
Demographics	
Age (years, mean ± SD)	73 ± 8.1
Male (N (%))	48 (65)
Female (N (%))	25 (35)
BMI (kg/m^2^, mean ± SD)	28.8 ± 4.8
CHA_2_DS_2_VASc Score (mean ± SD)	3.9 ± 0.9
HAS-BLED score (mean ± SD)	4.9 ± 0.8
Atrial fibrillation	
Paroxysmal (N (%))	36 (49)
Persistent (N (%))	7 (10)
Permanent (N (%))	31 (42)
Anticoagulation until day 45	
Vitamin K antagonist (N (%))	5 (7)
LMWH or NOAC (N (%))	18 (24)
DAPT (N (%))	18 (24)
Vitamin K antagonist or LMWH or NOAC + antiplatelet therapy (N (%))	33 (45)
Laboratory data	
Hemoglobin (mmol/dL; mean ± SD)	7.6 ± 1.6
eGFR (mL/min/1.73 m^2^; mean ± SD)	56.9 ± 23.4
BNP (pg/mL; *n* = 47, mean ± SD)	171.5 ± 110.9
Echocardiographic parameters	
LVEF ((%) *n* = 62)	58.1 ± 14.1
Left atrial diameter (mm, *n* = 50, mean ± SD)	46.3 ± 8.2
TRPG (mmHg, *n* = 33, mean ± SD)	35.2 ± 13.4
Procedural Data	
Occluder Size (mm, mean ± SD)	26.3 ± 3.8
Type of Occluder (N (%))	
Watchman device	66 (91.5)
LAmbre ™	6 (8.5)

BMI = Body mass index; CHA_2_DS_2_VASc Score = Congestive heart failure-Hypertension-Age-Diabetes-Stroke-Vascular disease-Age-Sex category Score; LMWH = Low molecular weight heparin; DAPT = Dual antiplatelet therapy; eGFR = Estimated glomerular filtration rate; BNP = Brain natriuretic peptide; LVEF = Left ventricular ejection fraction; TRPG = Tricuspid regurgitation peak gradient; SD = Standard deviation; N = number of patients; HAS-BLED = Hypertension, Abnormal renal/liver function, Stroke, Bleeding history or predisposition, Labile INR, Elderly (>65 years), Drugs/alcohol concomitantly; NOAC = Non-vitamin K antagonist oral anticoagulant.

**Table 2:** Parameters at baseline/45 days and their respective AUC-values regarding prediction of Type C after 6 months.

Parameters	AUC	CI (95%)	*p*-Value
Orifice area at baseline	0.78	0.61–0.95	0.019
Leakage size after 45 days	0.75	0.59–0.91	0.008
Mean compression rate after 45 days	0.47	0.27–0.68	0.79
Occluder size	0.77	0.60–0.93	0.005
CHA_2_DS_2_VASc score at baseline	0.56	0.37–0.75	0.57
HAS–BLED score at baseline	0.52	0.35–0.71	0.80
BNP at baseline	0.57	0.38–0.75	0.51
BNP after 45 days	0.61	0.42–0.79	0.31
Sphericity index at baseline	0.56	0.33–0.79	0.12

BNP = Brain natriuretic peptide; CHA_2_DS_2_VASc Score = Congestive heart failure-Hypertension-Age-Diabetes-Stroke-Vascular disease-Age-Sex category Score; HAS–BLED Score = Hypertension–Abnormal kidney or liver function–Stroke–Bleeding–Labile INR–Elderly–Drugs or alcohol; AUC = Area under the curve; CI = Confidence interval; *p* = level of significance.

**Table 3:** Parameters at baseline/45 days and their respective AUC-values regarding prediction of Type C after 6 months.

Parameters	Univariable		Multivariable	
	OR (CI)	*p*-Value	OR (CI)	*p*-Value
Atrial Fibrillation	0.73 (0.20–2.63)	0.627	-	-
Maximal ostial diameter in mm (2D)	1.19 (1.01–1.41)	0.043	0.98 (0.70–1.37)	0.898
Average ostial diameter in mm (2D)	1.30 (1.03–1.63)	0.026	1.04 (0.95–1.14)	0.411
3D perimeter in mm	1.06 (1.00–1.13)	0.043	1.22 (0.77–1.94)	0.402
3D orifice area in cm^2^	1.73 (0.98–3.05)	0.058	-	-
3D area derived diameter in mm	1.19 (0.99–1.44)	0.063	-	-
3D minimal ostial diameter in mm	1.18 (0.98–1.42)	0.076	-	-
3D maximal ostial diameter in mm	1.12 (0.97–1.29)	0.121	-	-
3D average ostial diameter in mm	1.19 (0.99–1.43)	0.061	-	-
Ostial sphericity index	0.29 (0.02–5.76)	0.418	-	-
Average LAA depth in mm	1.12 (0.96–1.30)	0.149	-	-
Maximal compression rate	0.94 (0.86–1.02)	0.154	-	-
Average compression rate	0.95 (0.86–1.04)	0.252	-	-
Implanted occluder device size in mm	1.23 (1.03–1.48)	0.023	1.01 (0.76–1.33)	0.963
Age in years	1.04 (0.96–1.12)	0.315	-	-
Sex	0.71 (0.23–2.12)	0.556	-	-
BMI in kg/m^2^	0.99 (0.88–1.11)	0.808	-	-
BSA in m^2^	1.20 (0.09–15.47)	0.887	-	-
eGFR in mL/min/kg	0.99 (0.97–1.01)	0.451	-	-
Hb in mmol/L	1.01 (0.71–1.44)	0.964	-	-
BNP in pg/mL	1.00 (0.99–1.00)	0.582	-	-
LVEF in%	0.99 (0.95–1.03)	0.588	-	-
LVEDD in mm	0.94 (0.84–1.05)	0.258	-	-
TR-PPG in mmHg	1.01 (0.96–1.07)	0.624	-	-

Hb, Hemoglobin; BMI, Body Mass Index; eGFR, estimated Glomerular Filtration Rate; BNP, Brain Natriuretic Peptide; LVEF, Left Ventricular Ejection Fraction; LVEDD, Left Ventricular End Diastolic Diameter; TR-PPG, Tricuspid Regurgitation Peak Pressure Gradient; OR, Odds Ratio; CI, confidence interval; LAA, Left atrial appendage.

**Table 4:** Sensitivity and specificity of Type C to predict. PDL after 45 days in transesophageal echocardiography.

Type C	Outcome	
	No PDL (*n* = 44)	PDL (*n* = 27)
No (*n* = 54)	44	10
Yes (*n* = 17)	0	17
Sensitivity	100%	
Specificity	63%	
PPV	81.5%	
NPV	100%	
PLR	2.7	

PDL, Peri device leak; *n*, absolute number; PPV, positive predictive value; NPV, negative predictive value; PLR, positive likelihood ratio.

## Data Availability

The data presented in this study are available on request from the corresponding author. The data are not publicly available due to local legal restrictions on data safety.

## Supplementary Materials

The following supporting information can be downloaded at: https://www.mdpi.com/article/10.3390/jcm11041059/s1, Video S1: Color Doppler of PDL; Video S2: LAA occluder before Contrast injection; Video S3: Device contrast enhancement.
